# *trans*-Diaqua­bis[(*E*)-3-(dimethyl­amino)-1-(2-pyrid­yl)prop-2-en-1-one-κ^2^ *N* ^1^,*O*]cobalt(II) dinitrate dihydrate

**DOI:** 10.1107/S1600536809016845

**Published:** 2009-05-14

**Authors:** Jian-Hong Bi

**Affiliations:** a Deparment of Chemistry and Chemical Engineering, Hefei Teachers College, Hefei, 230061, People’s Republic of China

## Abstract

In the title compound, [Co(C_10_H_12_N_2_O)_2_(H_2_O)_2_](NO_3_)_2_·2H_2_O, the Co^II^ ion, located on an inversion center, is *trans*-coordinated by two *N*,*O*-bidentate chelating (*E*)-3-(dimethyl­amino)-1-(2-pyrid­yl)prop-2-en-1-one ligands and by two water mol­ecules in a slightly distorted octa­hedral geometry. Inter­molecular O—H⋯O hydrogen bonds link the cations, anions and water mol­ecules into layers parallel to the *ac* plane. The crystal packing also exhibits weak inter­molecular C—H⋯O hydrogen bonds.

## Related literature

For the crystal structures of related complexes, see: Hu & Tian (2007 ▶); Li *et al.* (2005 ▶); Yan *et al.* (2004 ▶).
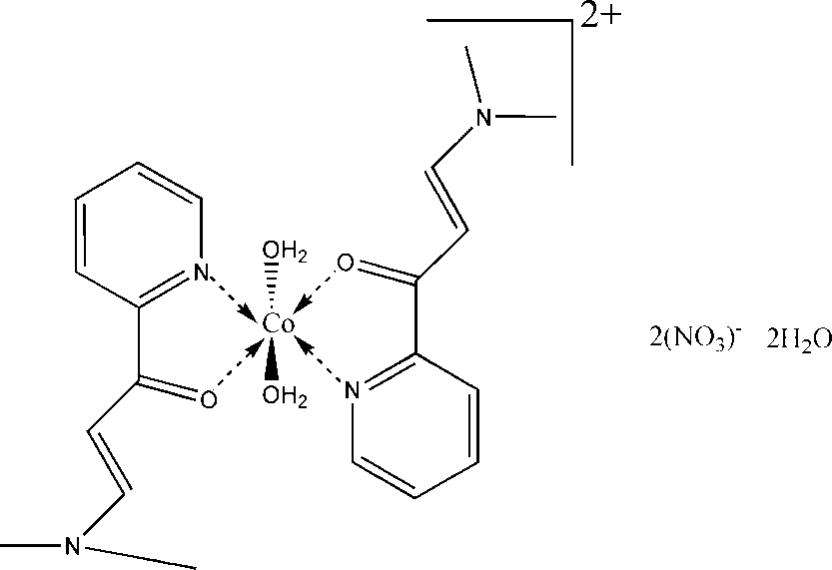


## Experimental

### 

#### Crystal data

[Co(C_10_H_12_N_2_O)_2_(H_2_O)_2_](NO_3_)_2_·2H_2_O
*M* *_r_* = 607.45
Triclinic, 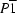

*a* = 7.8220 (19) Å
*b* = 8.646 (2) Å
*c* = 11.088 (3) Å
α = 98.439 (4)°
β = 101.239 (4)°
γ = 108.467 (4)°
*V* = 679.9 (3) Å^3^
*Z* = 1
Mo *K*α radiation
μ = 0.70 mm^−1^
*T* = 291 K
0.30 × 0.20 × 0.20 mm

#### Data collection

SMART CCD area-detector diffractometer
Absorption correction: multi-scan (*SADABS*; Bruker, 2000 ▶) *T*_min_ = 0.802, *T*_max_ = 0.876
3375 measured reflections
2342 independent reflections
2109 reflections with *I* > 2σ(*I*)
*R*_int_ = 0.024

#### Refinement

*R*[*F* ^2^ > 2σ(*F* ^2^)] = 0.052
*wR*(*F* ^2^) = 0.148
*S* = 1.08
2342 reflections
180 parameters
H-atom parameters constrained
Δρ_max_ = 0.50 e Å^−3^
Δρ_min_ = −0.41 e Å^−3^

### 

Data collection: *SMART* (Bruker, 2000 ▶); cell refinement: *SAINT* (Bruker, 2000 ▶); data reduction: *SAINT*; program(s) used to solve structure: *SHELXTL* (Sheldrick, 2008 ▶); program(s) used to refine structure: *SHELXTL*; molecular graphics: *SHELXTL*; software used to prepare material for publication: *SHELXTL*.

## Supplementary Material

Crystal structure: contains datablocks I, New_Global_Publ_Block. DOI: 10.1107/S1600536809016845/cv2560sup1.cif

Structure factors: contains datablocks I. DOI: 10.1107/S1600536809016845/cv2560Isup2.hkl

Additional supplementary materials:  crystallographic information; 3D view; checkCIF report

## Figures and Tables

**Table 1:** Hydrogen-bond geometry (Å, °)

*D*—H⋯*A*	*D*—H	H⋯*A*	*D*⋯*A*	*D*—H⋯*A*
O4—H4*B*⋯O6	0.85	2.22	2.764 (4)	121
O4—H4*C*⋯O1^i^	0.85	2.11	2.909 (6)	156
O4—H4*C*⋯O2^i^	0.85	2.42	3.173 (5)	149
O6—H6*A*⋯O3^ii^	0.85	2.44	3.011 (5)	125
O6—H6*C*⋯O3	0.85	2.23	2.987 (6)	148
C1—H1⋯O1^iii^	0.93	2.40	3.161 (5)	138
C4—H4*A*⋯O6^iv^	0.93	2.59	3.508 (4)	168
C9—H9*C*⋯O3^v^	0.96	2.53	3.351 (7)	144

Symmetry codes: (i) 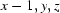; (ii) 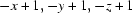; (iii) 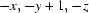; (iv) 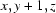; (v) 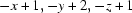.

**Fig. 1.**
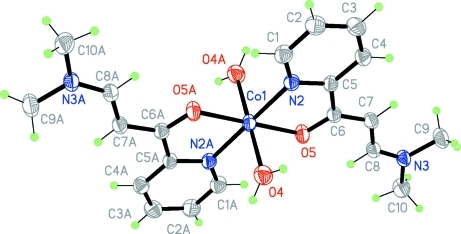
Molecular structure of the cation of the title compound, the anions and the free water molecules are omitted for clarity, showing 30% probability displacement ellipsoids and the atom-numbering [symmetry code: (A) -x, 1 - y, -z.

## supplementary crystallographic information

### Comment

The rational design and synthesis of coordinated complexes derived from 2-[3-(dimethylamino)prop-2-enoyl] pyridine have been of increasing interest recently in chemical research (Hu & Tian, 2007; Li *et al.*, 2005; Yan *et al.*, 2004). Here we report a new monomeric cobalt(II) complex, *viz.*the title compound, [Co(C_10_H_12_N_2_O)_2_(H_2_O)_2_](NO_3_)_2_(H_2_O)_2_.

The coordination geometry of the Co(II) center is shown in Fig.1. The Co(II) center adopts an octahedral coordination geomtry, where two N atoms and two O atoms from two ligands are in the equatorial plane while the apical positions are occupied by two water molecules. The asymmetric unit of the title compound contains a half of the complex, one crystalline water molecule and one nitrate counter-anion. The coordinated water molecules, crystalline water molecules and nitrate anions are involved in the hydrogen bonding interactions (Table 1).

### Experimental

All solvents and chemicals were of analytical grade and were used without further purification. For the synthesis of title compoud, a solution of ligand (0.2 mmol) and Co(NO_3_)_2_(0.1 mmol) in 50 ml me thanol was refluxed for 2 h, and then cooled to room temperature and filtered. Single crystals suitable for X-ray analysis were grown from the methanol solution by slow evaporation at room temperature in air. Anal. Calcd.for C_20_H_32_CoN_6_O_12_: C, 39.54; H, 5.31; N, 13.84. Found: C, 39.58; H,5.33; N, 13.79.

### Refinement

All hydrogen atoms were geomemetrically positioned (C—H 0.93–0.97 Å, O–H 0.85 Å) and refined as riding, with *U*_iso_(H)=1.2–1.5 *U*_eq_ of the parent atom.

### Crystal data

**Table:**

[Co(C_10_H_12_N_2_O)_2_(H_2_O)_2_](NO_3_)_2_·2H_2_O	*Z* = 1
*M**_r_* = 607.45	*F*(000) = 317
Triclinic, *P*1	*D*_x_ = 1.483 Mg m^−^^3^
Hall symbol: -P 1	Mo *K*α radiation, λ = 0.71073 Å
*a* = 7.8220 (19) Å	Cell parameters from 2398 reflections
*b* = 8.646 (2) Å	θ = 2.6–27.1°
*c* = 11.088 (3) Å	µ = 0.70 mm^−^^1^
α = 98.439 (4)°	*T* = 291 K
β = 101.239 (4)°	Block, purple
γ = 108.467 (4)°	0.30 × 0.20 × 0.20 mm
*V* = 679.9 (3) Å^3^	

### Data collection

**Table:**

SMART CCD area-detector diffractometer	2342 independent reflections
Radiation source: fine-focus sealed tube	2109 reflections with *I* > 2σ(*I*)
graphite	*R*_int_ = 0.024
φ and ω scan	θ_max_ = 25.0°, θ_min_ = 2.6°
Absorption correction: multi-scan (*SADABS*; Bruker, 2000)	*h* = −9→9
*T*_min_ = 0.802, *T*_max_ = 0.876	*k* = −6→10
3375 measured reflections	*l* = −13→11

### Refinement

**Table:**

Refinement on *F*^2^	Primary atom site location: structure-invariant direct methods
Least-squares matrix: full	Secondary atom site location: difference Fourier map
*R*[*F*^2^ > 2σ(*F*^2^)] = 0.052	Hydrogen site location: inferred from neighbouring sites
*wR*(*F*^2^) = 0.148	H-atom parameters constrained
*S* = 1.08	*w* = 1/[σ^2^(*F*_o_^2^) + (0.1051*P*)^2^] where *P* = (*F*_o_^2^ + 2*F*_c_^2^)/3
2342 reflections	(Δ/σ)_max_ = 0.008
180 parameters	Δρ_max_ = 0.50 e Å^−^^3^
0 restraints	Δρ_min_ = −0.41 e Å^−^^3^

### Special details

**Table:**

Experimental. The structure was solved by direct methods (Bruker, 2000) and successive difference Fourier syntheses.
Geometry. All e.s.d.'s (except the e.s.d. in the dihedral angle between two l.s. planes) are estimated using the full covariance matrix. The cell e.s.d.'s are taken into account individually in the estimation of e.s.d.'s in distances, angles and torsion angles; correlations between e.s.d.'s in cell parameters are only used when they are defined by crystal symmetry. An approximate (isotropic) treatment of cell e.s.d.'s is used for estimating e.s.d.'s involving l.s. planes.
Refinement. Refinement of *F*^2^ against ALL reflections. The weighted *R*-factor *wR* and goodness of fit *S* are based on *F*^2^, conventional *R*-factors *R* are based on *F*, with *F* set to zero for negative *F*^2^. The threshold expression of *F*^2^ > σ(*F*^2^) is used only for calculating *R*-factors(gt) *etc*. and is not relevant to the choice of reflections for refinement. *R*-factors based on *F*^2^ are statistically about twice as large as those based on *F*, and *R*- factors based on ALL data will be even larger.

### Fractional atomic coordinates and isotropic or equivalent isotropic displacement parameters (Å^2^)

**Table:**

	*x*	*y*	*z*	*U*_iso_*/*U*_eq_	
Co1	0.0000	0.5000	0.0000	0.0453 (2)	
O4	−0.0719 (3)	0.4009 (3)	0.1563 (2)	0.0642 (6)	
H4B	−0.0433	0.3211	0.1800	0.077*	
H4C	−0.1233	0.4574	0.1957	0.077*	
O5	0.2210 (3)	0.6910 (2)	0.1225 (2)	0.0543 (5)	
N2	−0.1181 (3)	0.6828 (3)	0.0345 (2)	0.0452 (5)	
N3	0.6416 (3)	1.0643 (3)	0.3847 (2)	0.0521 (6)	
C1	−0.2916 (4)	0.6719 (4)	−0.0148 (3)	0.0552 (7)	
H1	−0.3744	0.5719	−0.0684	0.066*	
C2	−0.3542 (5)	0.8023 (4)	0.0101 (3)	0.0619 (8)	
H2	−0.4774	0.7901	−0.0242	0.074*	
C3	−0.2303 (5)	0.9504 (4)	0.0866 (3)	0.0619 (8)	
H3	−0.2676	1.0416	0.1031	0.074*	
C4	−0.0494 (4)	0.9638 (4)	0.1394 (3)	0.0524 (7)	
H4A	0.0356	1.0633	0.1924	0.063*	
C5	0.0027 (4)	0.8274 (3)	0.1122 (2)	0.0432 (6)	
C6	0.1935 (4)	0.8238 (3)	0.1623 (3)	0.0437 (6)	
C7	0.3301 (4)	0.9602 (3)	0.2493 (3)	0.0491 (7)	
H7	0.3076	1.0585	0.2715	0.059*	
C8	0.4988 (4)	0.9492 (3)	0.3024 (3)	0.0488 (7)	
H8	0.5128	0.8477	0.2765	0.059*	
C9	0.6421 (6)	1.2306 (4)	0.4325 (4)	0.0769 (11)	
H9A	0.6102	1.2793	0.3630	0.115*	
H9B	0.7640	1.2996	0.4844	0.115*	
H9C	0.5526	1.2224	0.4817	0.115*	
C10	0.8112 (5)	1.0349 (5)	0.4342 (4)	0.0717 (10)	
H10A	0.8014	0.9257	0.3928	0.108*	
H10B	0.8292	1.0415	0.5232	0.108*	
H10C	0.9153	1.1179	0.4195	0.108*	
O1	0.6534 (6)	0.5229 (6)	0.2379 (3)	0.1422 (16)	
O2	0.8756 (5)	0.6226 (4)	0.3910 (4)	0.1396 (16)	
O3	0.6145 (7)	0.6168 (4)	0.4090 (4)	0.1368 (17)	
N1	0.7140 (4)	0.5912 (3)	0.3458 (3)	0.0624 (7)	
O6	0.2256 (4)	0.3681 (3)	0.3177 (3)	0.0876 (8)	
H6A	0.1895	0.3279	0.3778	0.131*	
H6C	0.3108	0.4638	0.3475	0.131*	

### Atomic displacement parameters (Å^2^)

**Table:**

	*U*^11^	*U*^22^	*U*^33^	*U*^12^	*U*^13^	*U*^23^
Co1	0.0425 (3)	0.0399 (3)	0.0469 (4)	0.0153 (2)	0.0049 (2)	−0.0024 (2)
O4	0.0786 (16)	0.0592 (13)	0.0621 (13)	0.0304 (12)	0.0249 (12)	0.0131 (11)
O5	0.0452 (11)	0.0460 (11)	0.0613 (12)	0.0195 (9)	−0.0002 (9)	−0.0080 (9)
N2	0.0417 (12)	0.0468 (12)	0.0447 (12)	0.0182 (10)	0.0061 (10)	0.0046 (10)
N3	0.0490 (14)	0.0405 (12)	0.0561 (15)	0.0129 (11)	0.0024 (11)	−0.0001 (11)
C1	0.0455 (16)	0.0598 (18)	0.0550 (17)	0.0183 (14)	0.0059 (13)	0.0070 (14)
C2	0.0496 (17)	0.071 (2)	0.067 (2)	0.0300 (16)	0.0088 (15)	0.0117 (17)
C3	0.064 (2)	0.065 (2)	0.070 (2)	0.0393 (17)	0.0205 (17)	0.0140 (16)
C4	0.0554 (17)	0.0525 (17)	0.0516 (17)	0.0249 (14)	0.0138 (14)	0.0053 (13)
C5	0.0461 (15)	0.0428 (14)	0.0396 (14)	0.0164 (12)	0.0111 (12)	0.0045 (11)
C6	0.0444 (15)	0.0428 (14)	0.0432 (14)	0.0166 (12)	0.0101 (12)	0.0063 (11)
C7	0.0504 (16)	0.0414 (14)	0.0525 (16)	0.0187 (12)	0.0079 (13)	0.0033 (12)
C8	0.0504 (16)	0.0395 (14)	0.0503 (16)	0.0127 (12)	0.0089 (13)	0.0043 (12)
C9	0.074 (2)	0.0486 (18)	0.087 (3)	0.0204 (17)	−0.005 (2)	−0.0101 (17)
C10	0.0522 (19)	0.065 (2)	0.081 (2)	0.0204 (16)	−0.0068 (17)	−0.0009 (18)
O1	0.121 (3)	0.212 (5)	0.060 (2)	0.042 (3)	−0.0073 (19)	0.011 (2)
O2	0.076 (2)	0.084 (2)	0.202 (4)	0.0161 (17)	−0.033 (2)	−0.020 (2)
O3	0.198 (4)	0.101 (2)	0.179 (4)	0.083 (3)	0.135 (4)	0.050 (2)
N1	0.0638 (18)	0.0571 (16)	0.0681 (18)	0.0249 (14)	0.0141 (15)	0.0154 (13)
O6	0.094 (2)	0.0814 (17)	0.0736 (17)	0.0298 (15)	0.0022 (15)	0.0041 (14)

### Geometric parameters (Å, °)

**Table:**

Co1—O5	2.0443 (19)	C3—H3	0.9300
Co1—O5^i^	2.0443 (19)	C4—C5	1.377 (4)
Co1—N2^i^	2.093 (2)	C4—H4A	0.9300
Co1—N2	2.093 (2)	C5—C6	1.499 (4)
Co1—O4^i^	2.136 (2)	C6—C7	1.389 (4)
Co1—O4	2.136 (2)	C7—C8	1.374 (4)
O4—H4B	0.8499	C7—H7	0.9300
O4—H4C	0.8500	C8—H8	0.9300
O5—C6	1.266 (3)	C9—H9A	0.9600
N2—C1	1.328 (4)	C9—H9B	0.9600
N2—C5	1.348 (3)	C9—H9C	0.9600
N3—C8	1.305 (4)	C10—H10A	0.9600
N3—C10	1.448 (4)	C10—H10B	0.9600
N3—C9	1.456 (4)	C10—H10C	0.9600
C1—C2	1.378 (5)	O1—N1	1.183 (4)
C1—H1	0.9300	O2—N1	1.191 (4)
C2—C3	1.369 (5)	O3—N1	1.192 (4)
C2—H2	0.9300	O6—H6A	0.8500
C3—C4	1.382 (4)	O6—H6C	0.8500
			
O5—Co1—O5^i^	180.0	C4—C3—H3	120.1
O5—Co1—N2^i^	101.59 (8)	C5—C4—C3	118.9 (3)
O5^i^—Co1—N2^i^	78.41 (8)	C5—C4—H4A	120.5
O5—Co1—N2	78.41 (8)	C3—C4—H4A	120.5
O5^i^—Co1—N2	101.59 (8)	N2—C5—C4	121.4 (3)
N2^i^—Co1—N2	180.00 (13)	N2—C5—C6	113.8 (2)
O5—Co1—O4^i^	90.60 (9)	C4—C5—C6	124.8 (2)
O5^i^—Co1—O4^i^	89.40 (9)	O5—C6—C7	122.7 (3)
N2^i^—Co1—O4^i^	91.84 (9)	O5—C6—C5	116.7 (2)
N2—Co1—O4^i^	88.16 (9)	C7—C6—C5	120.6 (2)
O5—Co1—O4	89.40 (9)	C8—C7—C6	119.7 (2)
O5^i^—Co1—O4	90.60 (9)	C8—C7—H7	120.1
N2^i^—Co1—O4	88.16 (9)	C6—C7—H7	120.1
N2—Co1—O4	91.84 (9)	N3—C8—C7	127.8 (3)
O4^i^—Co1—O4	180.0	N3—C8—H8	116.1
Co1—O4—H4B	124.2	C7—C8—H8	116.1
Co1—O4—H4C	111.2	N3—C9—H9A	109.5
H4B—O4—H4C	124.4	N3—C9—H9B	109.5
C6—O5—Co1	117.12 (18)	H9A—C9—H9B	109.5
C1—N2—C5	118.9 (2)	N3—C9—H9C	109.5
C1—N2—Co1	127.2 (2)	H9A—C9—H9C	109.5
C5—N2—Co1	113.84 (17)	H9B—C9—H9C	109.5
C8—N3—C10	122.0 (3)	N3—C10—H10A	109.5
C8—N3—C9	122.6 (3)	N3—C10—H10B	109.5
C10—N3—C9	115.5 (3)	H10A—C10—H10B	109.5
N2—C1—C2	122.9 (3)	N3—C10—H10C	109.5
N2—C1—H1	118.6	H10A—C10—H10C	109.5
C2—C1—H1	118.6	H10B—C10—H10C	109.5
C3—C2—C1	118.2 (3)	O1—N1—O2	117.4 (4)
C3—C2—H2	120.9	O1—N1—O3	121.2 (4)
C1—C2—H2	120.9	O2—N1—O3	121.2 (4)
C2—C3—C4	119.7 (3)	H6A—O6—H6C	109.5
C2—C3—H3	120.1		

Symmetry codes: (i) −*x*, −*y*+1, −*z*.

### Hydrogen-bond geometry (Å, °)

**Table:**

*D*—H···*A*	*D*—H	H···*A*	*D*···*A*	*D*—H···*A*
O4—H4B···O6	0.85	2.22	2.764 (4)	121
O4—H4C···O1^ii^	0.85	2.11	2.909 (6)	156
O4—H4C···O2^ii^	0.85	2.42	3.173 (5)	149
O6—H6A···O3^iii^	0.85	2.44	3.011 (5)	125
O6—H6C···O3	0.85	2.23	2.987 (6)	148
C1—H1···O1^i^	0.93	2.40	3.161 (5)	138
C4—H4A···O6^iv^	0.93	2.59	3.508 (4)	168
C9—H9C···O3^v^	0.96	2.53	3.351 (7)	144

Symmetry codes:  (ii) *x*−1, *y*, *z*; (iii) −*x*+1, −*y*+1, −*z*+1; (i) −*x*, −*y*+1, −*z*; (iv) *x*, *y*+1, *z*; (v) −*x*+1, −*y*+2, −*z*+1.
